# Modes of Gene Duplication Contribute Differently to Genetic Novelty and Redundancy, but Show Parallels across Divergent Angiosperms

**DOI:** 10.1371/journal.pone.0028150

**Published:** 2011-12-02

**Authors:** Yupeng Wang, Xiyin Wang, Haibao Tang, Xu Tan, Stephen P. Ficklin, F. Alex Feltus, Andrew H. Paterson

**Affiliations:** 1 Plant Genome Mapping Laboratory, University of Georgia, Athens, Georgia, United States of America; 2 Institute of Bioinformatics, University of Georgia, Athens, Georgia, United States of America; 3 College of Life Sciences, Hebei United University, Tangshan, Hebei, China; 4 Department of Plant Biology, University of Georgia, Athens, Georgia, United States of America; 5 Plant and Environmental Sciences, Clemson University, Clemson, South Carolina, United States of America; 6 Department of Genetics and Biochemistry, Clemson University, Clemson, South Carolina, United States of America; 7 Department of Crop and Soil Sciences, University of Georgia, Athens, Georgia, United States of America; 8 Department of Genetics, University of Georgia, Athens, Georgia, United States of America; UC Santa Barbara, United States of America

## Abstract

**Background:**

Both single gene and whole genome duplications (WGD) have recurred in angiosperm evolution. However, the evolutionary effects of different modes of gene duplication, especially regarding their contributions to genetic novelty or redundancy, have been inadequately explored.

**Results:**

In *Arabidopsis thaliana* and *Oryza sativa* (rice), species that deeply sample botanical diversity and for which expression data are available from a wide range of tissues and physiological conditions, we have compared expression divergence between genes duplicated by six different mechanisms (WGD, tandem, proximal, DNA based transposed, retrotransposed and dispersed), and between positional orthologs. Both neo-functionalization and genetic redundancy appear to contribute to retention of duplicate genes. Genes resulting from WGD and tandem duplications diverge slowest in both coding sequences and gene expression, and contribute most to genetic redundancy, while other duplication modes contribute more to evolutionary novelty. WGD duplicates may more frequently be retained due to dosage amplification, while inferred transposon mediated gene duplications tend to reduce gene expression levels. The extent of expression divergence between duplicates is discernibly related to duplication modes, different WGD events, amino acid divergence, and putatively neutral divergence (time), but the contribution of each factor is heterogeneous among duplication modes. Gene loss may retard inter-species expression divergence. Members of different gene families may have non-random patterns of origin that are similar in Arabidopsis and rice, suggesting the action of pan-taxon principles of molecular evolution.

**Conclusion:**

Gene duplication modes differ in contribution to genetic novelty and redundancy, but show some parallels in taxa separated by hundreds of millions of years of evolution.

## Introduction

Whole-genome duplications (WGDs) have occurred in the lineages of plants [1], animals [2], [3] and fungi [4], [5], with possible consequences including evolution of novel or modified gene functions [6], [7], [8], [9], and/or provision of “buffer capacity” [10], [11] or genetic redundancy that increases genetic robustness [12], [13], [14], [15], [16], [17]. Genome duplication may also increase opportunities for nonreciprocal recombination [18], [19], [20], permitting or causing duplicated genes to evolve in concert for a period of time. Rapid DNA loss and restructuring of low-copy DNA [21], [22], [23], [24], retrotransposon activation [25], [26], [27] and epigenetic changes [28], [29], [30], [31], [32], [33] following WGD may further provide materials for evolutionary change.

Genes may be duplicated by several mechanisms in addition to WGDs, which have been collectively referred to as small scale duplications [34] or single gene duplications [35], [36]. Tandem duplicates are consecutive in the genome while proximal duplicates are near one another but separated by a few genes. These two gene duplication modes are presumed to arise through unequal crossing over [36] or localized transposon activities [37]. Dispersed duplicates are neither adjacent to each other in the genome nor within homeologous chromosome segments [38]. Distant single gene transposition may explain the widespread existence of dispersed duplicates within and among genomes [36]. Distant single gene transposition duplication (referred to as distantly transposed duplication) may occur by DNA based or RNA based mechanisms [35]. DNA transposons such as packmules (rice) [39], helitrons (maize) [40], and CACTA elements (sorghum) [27] may relocate duplicated genes or gene segments to new chromosomal positions (referred to as DNA based transposed duplication). RNA based transposed duplication, often referred to as retrotransposition, typically creates a single-exon retrocopy from a multi-exon parental gene, by reverse transcription of a spliced messenger RNA. It is presumed that the retrocopy duplicates only the transcribed sequence of the parental gene, detached from the parental promoter. The new retrogene is often deposited in a novel chromosomal environment with new (i.e. non-ancestral) neighboring genes and, having lost its native promoter, is only likely to survive as a functional gene if a new promoter is acquired [41], [42].

Classical population genetic theory suggests that a likely consequence of gene duplication is reversion to single copy (singleton), unless at least one gene copy evolves new function [8]. More recently, the subfunctionalization model, which proposes that duplicated gene copies might both be retained if they partition the functions of the ancestral gene between them, has described an important modification of the classical model [9], [43]. Some studies also show evidence to support the value of genetic redundancy *per se* [10], [12], [13], [14], [15], [16], [17], [44], [45] or dosage balance [34], [46], [47], [48].

The angiosperms (flowering plants) are an outstanding model in which to elucidate the consequences of gene duplication. All angiosperms are now thought to be paleopolyploids [49], many of which underwent multiple WGDs [50], [51]. Traces of past WGDs can often be detected from pairwise syntenic alignments through software such as ColinearScan [52] and multiple alignments using MCScan [53]. Arabidopsis, selected as the first angiosperm genome to be sequenced due to its small genome size and minimal DNA sequence duplication, has experienced two ‘recent’ WGDs, i.e. since its divergence from other members of the Brassicales clade (α and β), and a more ancient triplication (γ) shared with most if not all eudicots [49], [51], [53]. Likewise, rice appears to have experienced at least two WGDs, one shared with most if not all cereals (ρ), and another more ancient event (σ) [54]. Single gene duplications in angiosperms are also widespread [36], [55], [56].

One avenue for systematic investigation of functional divergence between duplicate genes is comparison of their spatiotemporal expression profiles, comparing degrees of divergence with proxies of duplication age such as synonymous substitution rates (Ks) between duplicate genes. In Arabidopsis, the rate of protein sequence evolution is asymmetric in >20% of duplicate pairs and functional diversification of surviving duplicate genes has been proposed to be a major feature of the long-term evolution of polyploids [57]. Arabidopsis genes created by large-scale duplication events are more evolutionarily conserved in gene expression than those created by small-scale duplication or those that do not lie in duplicate segments, and the time since duplication is correlated with functional divergence of genes [58]. Further, there may be also a strong positive correlation between expression divergence and non-synonymous mutation (Ka) in Arabidopsis, and the different modes (segmental, tandem and dispersed) of duplication may affect patterns of expression divergence [38]. Arabidopsis duplicated genes show greater expression diversity than singleton genes across closely related species and allopolyploids [59]. In rice, expression correlation is significantly higher for gene pairs from WGDs or tandem duplications than dispersed duplications, and expression divergence is closely related to divergence time [60].

Though many studies have investigated the functional divergence and retention of duplicate genes, conclusions are often contradictory, e.g. gene retention has been attributed to either neofunctionalization [6], [7] or genetic redundancy [12], [13], [14], [15], [16], [17], and expression divergence between duplicate genes has been suggested to be either time dependent [58], [60] or selection dependent [38]. The fates of duplicate genes may be influenced by different modes of gene duplication, which have been suggested to retain genes in a biased manner [36]. With much richer expression and annotation data available now than for most prior studies, and improved ability to discern various mechanisms of gene duplication, we find merit in re-examining some existing hypotheses and exploring some new hypotheses regarding the consequences of gene duplication. Here, we related multiple types of genomic data to gene expression divergence in two angiosperm species, Arabidopsis and Oryza (rice), to formally test possible evolutionary patterns (hypotheses). A far richer volume of analyzed microarray data than was available in prior studies improves the robustness of statistical analyses.

## Results

**Figure 1:**
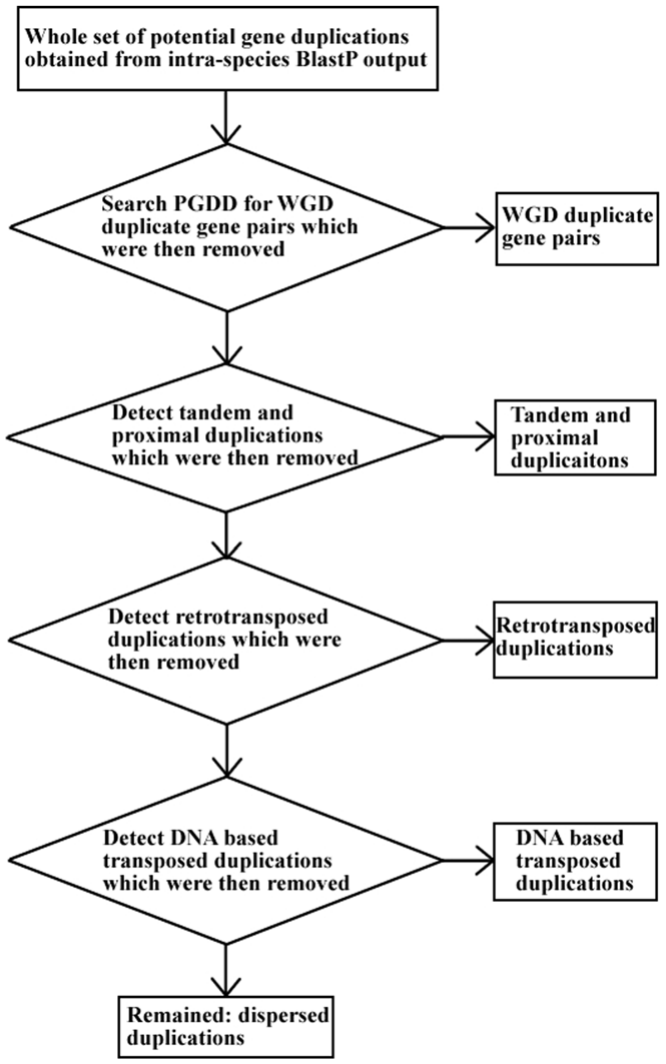
Flowchart of the procedure for classifying gene pairs based on mode of duplication.

A total of 4,566 Affymetrix Arabidopsis Genome ATH1 Arrays and 508 Affymetrix GeneChip Rice Genome Arrays were used to generate the expression profiles of 22,810 Arabidopsis genes and 27,910 rice genes. We classified gene duplications into six modes: WGD, tandem, proximal, DNA based transposed, retrotransposed and dispersed duplication, according to the procedure shown in Figure 1 and described in methods. Note that in this study, a gene may have up to five potential duplication relationships, depending on the number of BLASTP hits. For WGD duplicates, redundant duplication relationships were removed using co-linearity restrictions. If a gene was created by single gene duplications, all possible duplication relationships were considered. However, redundant duplication relationships in single gene duplications did not enlarge the gene set created by each duplication mode. In a distantly transposed duplication, one duplicate gene is the parental (ancestral) copy while the other is the transposed (derived) copy, at a novel locus. Dispersed duplications, which we cannot attribute to specific mechanisms, are regarded as a control group. The number of pairs of duplicate genes and number of unique genes (i.e. number of created genes) in each mode of duplication is summarized in Table 1. A total of 2,981 α, 1,161 β and 417 γ WGD duplicate pairs in Arabidopsis; and 1,712 ρ and 568 γ WGD duplicate pairs in rice, have expression profiles. In this study, the degree of similarity between the expression profiles of a pair of genes across all experiments is measured by the Pearson's correlation coefficient (*r*). To express in positive values the evolution of gene expression between duplicates or orthologs, we use the term “expression divergence”, measured by 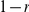 [61], [62].

**Table 1:** Numbers of pairs of duplicate genes and unique genes in each mode of gene duplication.

Mode of duplication	Number of pairs of duplicate genes (number of those having complete expression profiles)		Number of unique genes (number of those having expression profiles)	
	Arabidopsis	Rice	Arabidopsis	Rice
WGD	6,572 (4,979)	3,593 (2,530)	9,455 (8,089)	5,723 (4,829)
Tandem	2,055 (1,055)	1,741(947)	1,586 (977)	2,948 (2,116)
Proximal	3,113 (1,456)	3,816 (1,990)	669 (379)	1,038 (714)
DNA based transposed	6,367 (4,088)	8,061 (5,225)	2,230 (1,572)	2,948 (2,116)
Retro- transposed	497 (300)	940 (681)	271 (1,71)	491 (391)
Dispersed	34,887 (26,127)	30,574 (21,385)	7,411 (6,182)	8,313 (6,960)

### Gene duplication modes contribute differentially to genetic novelty and redundancy

**Figure 2:**
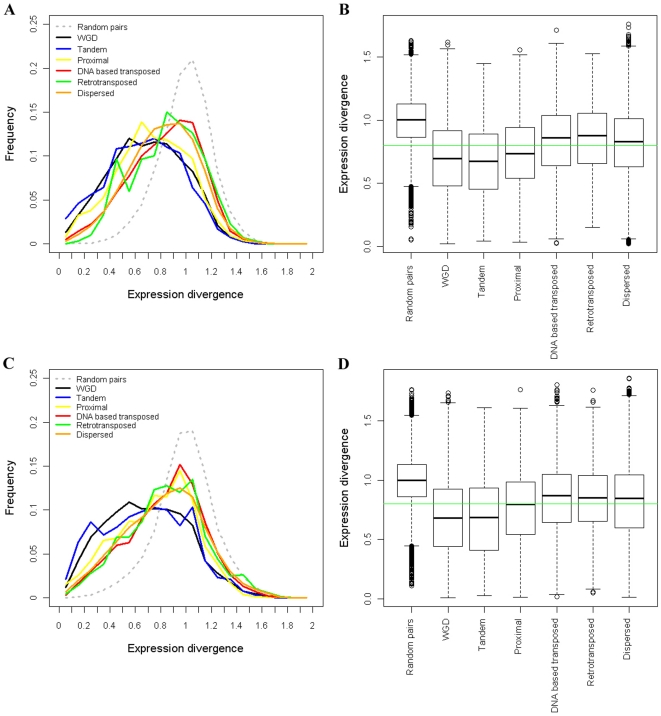
Comparison of expression divergence among different modes of gene duplication. (A) Comparison of distributions of expression divergence in Arabidopsis. (B) Comparison of levels of expression divergence in Arabidopsis. (C) Comparison of distributions of expression divergence in rice. (D) Comparison of levels of expression divergence in rice. Green lines in (B, D) indicate average expression divergence across duplication modes.

Expression divergence between duplicate genes was compared across modes of duplication (Figure 2). The trends of expression divergence between duplicates in Arabidopsis and rice are very similar: DNA based transposed duplication≈retrotransposed duplication > dispersed duplication > proximal duplication > WGD≈tandem duplication (both ANOVA model involving all duplication modes and Tukey's HSD test between adjacent duplication modes are significant at α = 0.05). Although retrotransposed duplications have a little higher average expression divergence than DNA based transposed duplications, the difference is not significant (*P*-value>0.05). WGDs result in a little higher expression divergence than tandem duplications in Arabidopsis but the difference is not significant in rice.

Despite the relatively fast evolution of gene expression shown by distantly transposed duplications, a tendency toward co-expression between genes duplicated by all modes can be observed by comparison with 10,000 randomly selected gene pairs (Figure 2). Furthermore, we used r<0.371 and r<0.621 (95% quantile of the *r* values obtained from random gene pairs) as criteria for determining that two duplicate genes have diverged in expression in Arabidopsis and rice respectively [57], [63]. The proportions of divergent expression between genes duplicated by different modes are shown in Table 2. All these data suggest that the extent of expression divergence of retained duplicates is affected by the duplication mechanism: WGD and tandem duplicates are more likely to maintain their original expression patterns, proximal duplications show intermediate divergence, and distantly transposed duplications tend to have the biggest changes of gene expression profiles.

**Table 2:** Proportion of divergent gene expression between duplicates in each mode of gene duplication.

Species	WGD	Tandem duplication	Proximal duplication	DNA based transposed duplication	Retrotransposed duplication	Dispersed duplication
Arabidopsis	0.577	0.555	0.644	0.759	0.767	0.759
Rice	0.813	0.780	0.865	0.916	0.921	0.904

Computationally, genetic redundancy may be inferred from simultaneous conservation in protein sequences that determine molecular functions, and expression patterns which determine biological processes [64], [65]. WGD and tandem duplicates tend to be simultaneously conserved in protein sequences (using 25% quartile of Ka of all duplicate pairs, i.e. <0.329 in Arabidopsis and <0.383 in rice, as criteria) and in gene expression (using 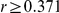 in Arabidopsis and 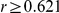 in rice as criteria), while distantly transposed and dispersed duplicates have a random association (assuming that conservation in protein sequences and gene expression were independent in the pooled duplicate genes) between these parameters, and proximal duplicates fall in between (Table 3).

**Table 3:** Proportion of conservation in both protein sequences and gene expression between duplicates in each mode of gene duplication.

Species	WGD	Tandem duplication	Proximal duplication	DNA based transposed duplication	Retro-transposed duplication	Dispersed duplication	Expected
Arabidopsis	0.335	0.328	0.231	0.071	0.051	0.038	0.071
Rice	0.140	0.170	0.099	0.027	0.023	0.021	0.041

**Figure 3:**
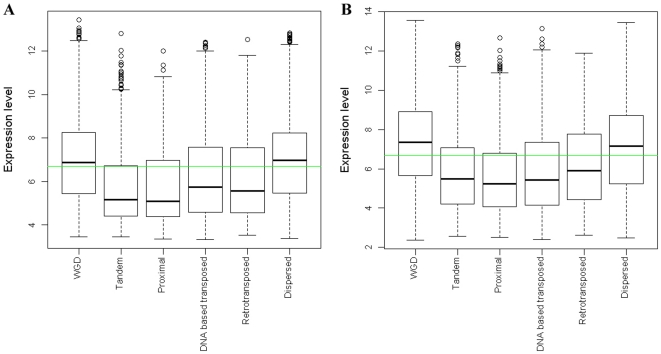
Comparison of expression levels between genes created by different duplication modes. (A) Comparison of expression levels between Arabidopsis genes created by different duplication modes. (B) Comparison of expression levels between rice genes created by different duplication modes. Green lines indicate average expression levels.

Expression levels differ between the genes created by different duplication modes (Figure 3). WGD and dispersed duplicates have higher gene expression levels than tandem, proximal and distantly transposed duplications (2-sample *t*-tests are significant at α = 0.05). The higher expression of WGD duplicates is consistent with their retention due to dosage amplification, a theory which has been proven in yeast [47], [66], [67]. Potentially transposon mediated gene duplications including tandem, proximal and distantly transposed duplications tend to be associated with lower gene expression levels than other duplication modes (Figure 3). Dispersed duplication, with unclear genetic mechanisms so far, is associated with gene expression levels comparable to WGD.

### Expression divergence following polyploidy

Since its divergence from other Brassicales, Arabidopsis experienced two WGDs (α and β), while sharing a more ancient genome triplication (γ) with all rosids and perhaps all eudicots [49], [51], [53]. Rice has experienced two WGDs: the ρ event shared with all Poaceae, and the more ancient σ event [54]. Although expression divergence has been compared between WGD and single gene duplications [38], [58], [60], the combinational effects of different WGD events on expression divergence have not been addressed. We propose that WGD events themselves, together with the subsequent ‘adaptation’ of the resulting genome to the newly-duplicated state, may accelerate evolution, contributing to variation in expression divergence sometimes attributed to time (usually measured by Ks) alone [58], [60].

**Figure 4:**
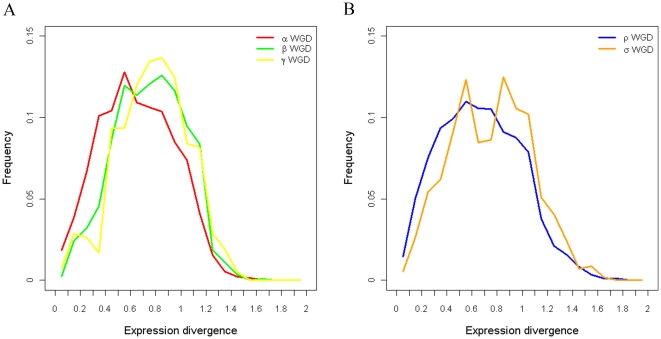
Comparison of distributions of expression divergence among different WGD events. (A) Comparison of distributions of expression divergence among different Arabidopsis WGD events. (B) Comparison of distributions of expression divergence among different rice WGD events. α, β and ρ were relatively recent WGD events, while γ and σ were more ancient WGD events.

To further investigate the combinational effects of multiple WGD events, we compared the expression divergence of duplicates from different WGD events (Figure 4). Not surprisingly, expression divergence between the WGD duplicates of more ancient events tends to be larger: γ duplicates > β duplicates > α duplicates in Arabidopsis, and σ duplicates > ρ duplicates in rice (both ANOVA model involving all WGD events and Tukey's HSD test between adjacent WGD events are significant at α = 0.05). Next, we fitted a curve between expression divergence and Ks for each WGD event using a smooth spline with 10 degrees of freedom available in R packages (Figure 4). We found no significant correlation between expression divergence and Ks within the more ancient Arabidopsis β duplicates (*r* = 0.036, *P*-value = 0.241) or γ duplicates (*r* = −0.008, *P*-value = 0.883), or rice σ duplicates (*r* = 0.045, *P*-value = 0.307) but correlations are significant within the most recent Arabidopsis α duplicates (*r* = 0.126, *P*-value = 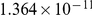) and rice ρ duplicates (*r* = 0.105, *P*-value = 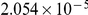). Further, we conducted a power analysis for these correlations. We found that at α = 0.05, the non-significant correlations (β, γ and σ duplicates) did not have higher power than conventionally desired (>0.8) while significant correlations (α and ρ duplicates) had power greater than 0.98, confirming that the relationship between expression divergence and Ks differs among different WGD events.

**Figure 5:**
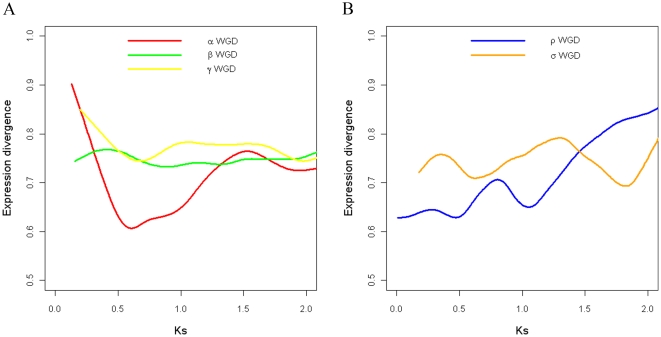
Fitted smooth spline curves between expression divergence and Ks for different WGD events. (A) Fitted smooth spline curves between expression divergence and Ks for different Arabidopsis WGD events. (B) Fitted smooth spline curves between expression divergence and Ks for different rice WGD events. α, β and ρ were relatively recent WGD events, while γ and σ were more ancient WGD events.

WGD events themselves influence gene expression divergence, with more ancient WGD duplicated genes likely to have greater expression divergence than more recent duplications, even if both have similar Ks (Figure 5). To support this hypothesis statistically, we coded the α, β and γ events by 1, 2 and 3 in Arabidopsis and the ρ and σ events by 1 and 2 in rice. Then different linear regression models of expression divergence on Ks and/or WGD codes were fit in Arabidopsis and rice respectively. All regression models and their coefficients were statistically significant. For both Arabidopsis and rice, the model which counts both Ks and the number of WGD events that duplicate genes underwent results in the highest adjusted *R* ^2^ and lowest Akaike information criterion (AIC) (Table 4) with significant nonzero slopes of all coefficients, supporting the hypothesis that WGD events themselves, in addition to Ks, can lead to increased expression divergence between duplicates.

**Table 4:** Linear regression of expression divergence (*d*) on Ks and WGD events (*W*).

Regression model	Coefficient (*P*-value)			Adjusted *R^2^*	AIC
	*a*	*b_1_*	*b_2_*		
Arabidopsis					
*d* = *a*+*b_1_*·*Ks*	0.593 (<2.2×10^−16^)	0.079 (<2.2×10^−16^)	-	0.027	−10706.164
*d* = *a*+*b_2_*·*W*	0.577 (<2.2×10^−16^)	-	0.074 (<2.2×10^−16^)	0.027	−10706.330
*d* = *a*+*b_1_*·*Ks+b_2_*·*W*	0.559 (<2.2×10^−16^)	0.050 (1.15×10^−8^)	0.047 (1.05×10^−8^)	0.034	−10736.930
Rice					
*d* = *a*+*b_1_*·*Ks*	0.624 (<2.2×10^−16^)	0.081 (1.84×10^−7^)	-	0.012	−4913.4477
*d* = *a*+*b_2_*·*W*	0.587 (<2.2×10^−16^)	-	0.079 (8.28×10^−7^)	0.011	−4916.3561
*d* = *a*+*b_1_*·*Ks+b_2_*·*W*	0.557 (<2.2×10^−16^)	0.063 (1.44×10^−4^)	0.058 (6.82×10^−4^)	0.017	−4925.9138

Selection after WGD events may constrain expression divergence of some duplicates. To examine this question, we studied the 25% of WGD duplicate pairs with most conserved expression at each WGD event. At a *P*-value threshold of 0.05 by Fisher's exact test (corrected for multiple tests), specific GO terms/Pfam domains were associated with conserved expression at each WGD event, and some recurred across different WGD events, e.g. transcription factor activity (GO:0003700) and ribosome (GO:0005840) for Arabidopsis α and γ and rice ρ events; protein biosynthesis (GO:0006412) for Arabidopsis α and β and rice ρ events (Table S1). In contrast, WGD duplicates with divergent expression (25% of pairs with highest *d* values at each event) showed little or no enrichment of specific GO terms/Pfam domains and functional terms did not recur between different WGD events.

### Expression divergence between Arabidopsis and rice

**Figure 6:**
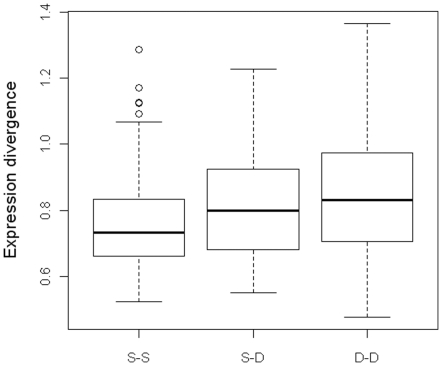
Comparison of expression divergence between different types of Arabidopsis-rice orthologs: singleton-singleton (S-S), singleton-duplicate (S-D) and duplicate-duplicate (D-D).

In that most angiosperms share most genes, changes in expression may be fundamental to angiosperm biodiversity. Previous studies have associated duplicated genes with greater expression diversity than singletons in closely related species of both animals [68] and plants [59]. However, it has been difficult to extend such comparisons to more distant species such as Arabidopsis, a eudicot, and rice, a monocot, due to greater difficulty discerning orthology or paralogy. To facilitate the comparison of gene expression data generated by different microarray platforms, we adopted a conceptual framework of comparing co-expression patterns across species [69] (see Methods). Further, we restricted our study to 2,012 gene pairs suggested both by DNA sequence similarity and by synteny/collinearity to be orthologs between Arabidopsis and rice, downloaded from the PGDD database [51], [53]. The comparison of expression divergence between different types of orthologs shows the following trend: duplicate-duplicate>singleton-duplicate>singleton-singleton (Figure 6), with *P*-values of 0.049 between duplicate-duplicate and singleton-duplicate and 0.010 between singleton-duplicate and singleton-singleton using two-sample *t*-tests. This finding supports that singletons are more conserved in expression than duplicated genes, consistent with the hypothesis that one consequence of gene duplication is increased expression diversity.

### Expression divergence may be correlated with both Ks and Ka

Divergence in coding sequences can be denoted by Ks, which indicates putatively-neutral mutations that are synonymous at the amino acid level, or by Ka, which indicates altered amino acids suggestive of the action of selection on gene function. The correlations between expression divergence and coding sequence divergence in angiosperms have been widely discussed [38], [58], [60] but conclusions were inconsistent: Casneuf et al. and Li et al. suggested that Ks is closely correlated with gene expression divergence, while Ganko et al. found little correlation. Since microarray data contain a high level of noise and previous studies often relied on small sets of microarray data or only one species, our analysis of “all arrays” and two highly-divergent species may have broader inference space.

**Figure 7:**
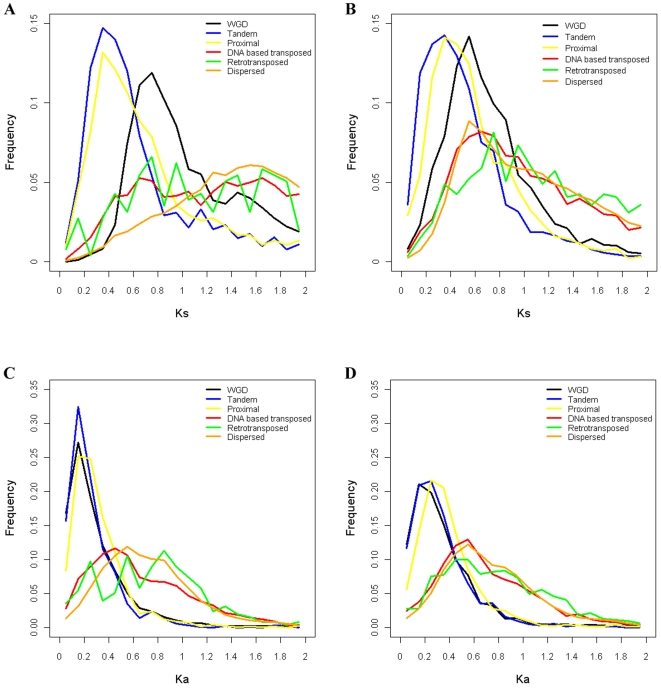
Comparison of Ks and Ka distributions for gene pairs duplicated by different modes. (A) Comparison of Ks distributions in Arabidopsis. (B) Comparison of Ks distributions in rice. (C) Comparison of Ka distributions in Arabidopsis. (D) Comparison of Ka distributions in rice.

The distributions of Ka or Ks differ markedly for different gene duplication modes, but are relatively consistent in Arabidopsis and rice (Figure 7). Tandem/proximal and WGD duplicates have qualitatively lower Ks (putatively reflecting younger age) than distantly transposed (DNA and RNA) or dispersed duplicates, the distinction being much clearer in the small genome of Arabidopsis (Figure 7A) than the 3× larger and more repeat-rich genome of rice (Figure 7B). Within these qualitative distinctions, quantitative differences among the categories are also evident and largely consistent, with relative Ks (putatively age) of duplications following the trend of: dispersed > distantly transposed > WGD > proximal > tandem (both ANOVA model involving all duplication modes and Tukey's HSD test between adjacent duplication modes are significant at α = 0.05). Retrotransposed duplicates differ slightly in the two taxa, being similar to DNA based transposed duplicates in Arabidopsis, and to dispersed duplicates in rice. The trend of Ka shows the same qualitative distinction as that of Ks (Figure 7C and 7D), but differing in the quantitative trend with amino-acid altering mutation frequencies being retrotransposed > dispersed > DNA based transposed > proximal≈WGD≈tandem (both ANOVA model involving all duplication modes and Tukey's HSD test between adjacent duplication modes are significant at α = 0.05). WGD duplicates are more functionally constrained, with higher Ks but equal or lower Ka than proximal duplicates. These data do not show the conventional L-shaped distribution for dispersed and distantly transposed duplicates, because the filters employed in gene selection focus this analysis only on genes that have survived a long time, implying that the genes serve important functions.

Relationships between coding sequence divergence and expression divergence are heterogeneous, and differ among gene duplication modes. For WGD duplicates, expression divergence is significantly correlated with both Ka and Ks in both Arabidopsis and rice, although the strength of the correlations is progressively weaker for more ancient duplications and in some cases reaches non-significance (Table 5). Expression divergence is also significantly correlated with both Ka and Ks among proximal duplicates. Tandem duplicates differ in the two taxa, with those of rice resembling WGD genes with expression divergence significantly correlated with both Ka and Ks, and those of Arabidopsis resembling distantly transposed duplications with marginal and sometimes non-significant correlation.

**Table 5:** Correlations between expression divergence (*d*) and coding sequence divergence.

Types of homologs	Number of valid gene pairs	Pearson correlation (*P*-value) between *d* and	
		Ka	Ks
Arabidopsis duplicates			
WGD	4,682	0.238 (<2.2×10^−16^)	0.176 (<2.2×10^−16^)
α	2,858	0.247 (<2.2×10^−16^)	0.126 (1.364×10^−11^)
β	1,068	0.146 (1.791×10^−6^)	0.036 (0.241)
γ	371	0.060 (0.253)	−0.008 (0.883)
Tandem	1,033	0.015 (0.635)	0.115 (2.137×10^−4^)
Proximal	1,426	0.057 (0.032)	0.113 (1.891×10^−5^)
DNA based transposed	3,662	0.052 (0.002)	0.023 (0.173)
Retrotransposed	257	0.042 (0.504)	0.142 (0.023)
Dispersed	23,360	0.046 (3.243×10^−12^)	0.047 (1.087×10^−12^)
Rice duplicates			
WGD	2,390	0.112 (4.006×10^−8^)	0.112 (3.984×10^−8^)
ρ	1,630	0.099 (6.519×10^−5^)	0.105 (2.054×10^−5^)
σ	521	0.059 (0.177)	0.045 (0.307)
Tandem	919	0.091 (0.006)	0.087 (0.008)
Proximal	1,898	0.084 (2.389×10^−4^)	0.095 (3.604×10^−5^)
DNA based transposed	4,687	0.056 (1.126×10^−4^)	0.017 (0.255)
Retrotransposed	613	0.008 (0.839)	0.037 (0.361)
Dispersed	19,397	0.037 (2.225×10^−7^)	0.017 (0.021)
Arabidopsis-rice orthologs	1,290	0.108 (9.468×10^−5^)	0.003 (0.901)

While age and functional divergence are more closely related to expression divergence in WGD genes than those resulting from other duplication modes, this does not reflect a lack of expression divergence among other gene duplicates. Indeed, proximal duplication is associated with higher expression divergence than WGD, despite its smaller average Ks. Likewise, DNA based transposed duplication is associated with higher expression divergence than dispersed duplication, despite smaller Ks (Table 6).

**Table 6:** Comparisons of expression divergence and Ks between WGD and proximal duplication, and between dispersed and DNA based transposed duplication.

Duplication modes	Arabidopsis		Rice	
	Mean d (*P*-value by *t*-test)	Mean Ks (*P*-value by *t*-test)	Mean d (*P*-value by *t*-test)	Mean Ks (*P*-value by *t*-test)
WGD vs Proximal	0.690 vs 0.731 (2.912×10^−6^)	1.162 vs 0.816 (<2.2×10^−16^)	0.690 vs 0.758 (1.47×10^−12^)	0.759 vs 0.619 (<2.2×10^−16^)
Dispersed vs DNA based transposed	0.813 vs 0.825 (0.019)	1.710 vs 1.490 (<2.2×10^−16^)	0.821 vs 0.825 (0.490)	1.169 vs 1.490 (<2.2×10^−16^)

**Figure 8:**
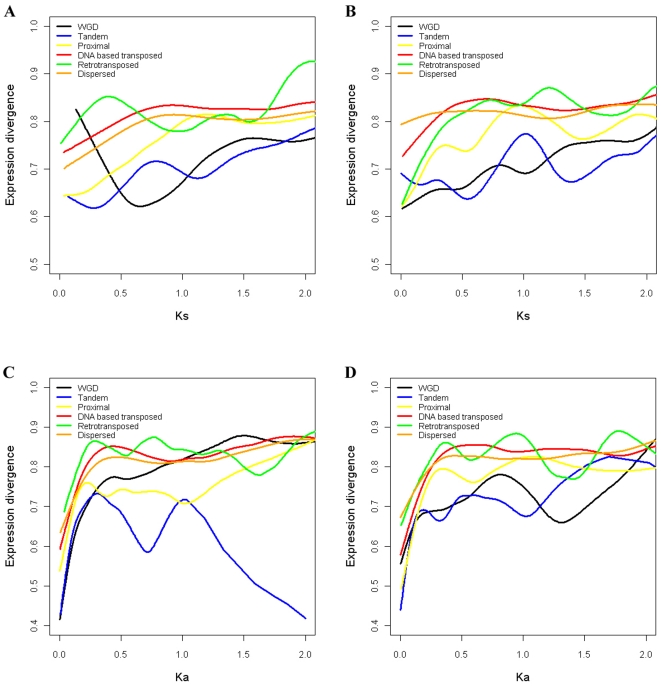
Fitted smooth spline curves between expression divergence and Ks or Ka for different modes of gene duplication. (A) Fitted smooth spline curves between expression divergence and Ks in Arabidopsis. (B) Fitted smooth spline curves between expression divergence and Ks in rice. (C) Fitted smooth spline curves between expression divergence and Ka in Arabidopsis. (D) Fitted smooth spline curves between expression divergence and Ka in rice.

In partial summary, expression divergence between duplicate genes may be affected by duplication modes, as well as by the ‘age’ (Ks) of the duplicated genes, i.e. gene expression divergence may differ among duplication modes at the same Ks or Ka levels. To further validate this claim, we fit a smooth spline curve between expression divergence and Ks or Ka for each duplication mode (Figure 8). While these curves fluctuate markedly, at fixed Ks or Ka levels distantly transposed duplications (for example) are generally associated with higher expression divergence between duplicates than WGD or tandem duplications.

### DNA methylation of the promoter regions has little impact on expression divergence

Epigenetic mechanisms such as DNA methylation have been suggested to potentially differentiate newly arisen duplicate genes [32], [70] as well as orthologous genes across closely related species [59]. Transcriptional silencing has often been associated with DNA methylation in promoter regions [71], [72]. Using data on genome-wide DNA methylation status for both Arabidopsis and rice [73], we examined whether DNA methylation status in promoter regions is related to expression divergence between duplicates or between orthologs. This comparison carries an inherent assumption that methylation patterns are relatively static and generally apply to all of the microarray studies. A gene promoter region was considered to be methylated if two or more adjacent probes are methylated within the region [72]. Proportions of pairs of duplicates that differ in DNA methylation status in promoter regions, separated by gene duplication modes, are summarized in Table 7. Distantly transposed duplications appear somewhat more likely to differ in DNA methylation status than other duplication modes. However, the duplicate genes that differ in DNA methylation status in promoter regions do not have more divergent expression than those that have the same DNA methylation status, within any duplication mode (negative data are not shown). Likewise, different methylation status among orthologs also showed no significant relationship to expression divergence, although we confirmed that singletons are a little more likely to be methylated in promoter regions than duplicates (Table 8), as proposed by others [59]. These analyses suggest that the mechanisms by which DNA methylation status affects expression divergence between homologous genes may be complicated, and direct association may not be informative for unraveling such mechanisms.

**Table 7:** Proportion of pairs of duplicates that have changed DNA methylation status in promoter regions.

Species	WGD	Tandem duplication	Proximal duplication	DNA based transposed duplication	Retrotransposed duplication	Dispersed duplication
Arabidopsis	0.303	0.290	0.309	0.387	0.347	0.318
Rice	0.357	0.417	0.404	0.416	0.447	0.385

**Table 8:** Proportion of genes that are methylated in promoter regions.

Species	Singletons	Duplicate genes
Arabidopsis	0.185	0.157
Rice	0.224	0.217

### Gene family members may have non-random patterns of origin

The diversity of gene duplication mechanisms and patterns of gene expression divergence raise questions about how gene families expand and how their members have been retained in the history of evolution. WGD duplicates are differentially retained across different gene functional classifications [10], [34], [57], [74]. However, we suggest that gene families may be more informative units than functional terms for investigating patterns of gene origin, as duplication relationships in gene families are clearer. Based on our findings above, both functional divergence and redundancy may contribute to retention of duplicate genes. Furthermore, because the degrees of functional diversification are not equal across gene families and gene duplication modes add additional heterogeneity to patterns of functional divergence, it is possible that gene family members may have non-random patterns of origin, e.g. the gene families with high functional diversification may be enriched with distantly transposed duplications while those families contributing to genetic redundancy are likely to be enriched with WGD duplications.

**Figure 9:**
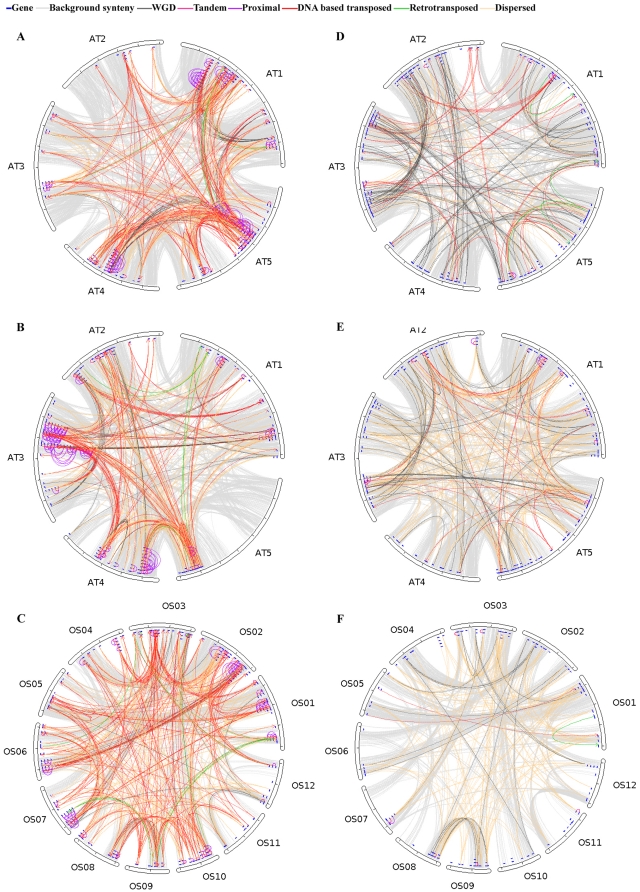
Gene duplication modes among the members of selected gene families. (A) Arabidopsis disease resistance gene homologs. (B) Arabidopsis Cytochrome P450 gene family. (C) Rice Cytochrome P450 gene family. (D) Arabidopsis cytoplasmic ribosomal gene family. (E) Arabidopsis C2H2 zinc finger gene family. (F) Rice C2H2 zinc finger gene family. Different gene duplication modes are indicated by different colors.

To examine these questions, we investigated the gene duplication modes of 126 Arabidopsis and 24 rice published gene families of 10 or more genes, available at TAIR (http://www.arabidopsis.org/) and Michigan State University (http://rice.plantbiology.msu.edu/) respectively. By using Bonferroni-corrected Fisher's exact test, we found that 64 (50.8%) Arabidopsis gene families and 19 (79.2%) rice gene families are enriched for at least one gene duplication mode at α = 0.05 (Table S2). For example, DNA based transposed duplications are enriched in disease resistance gene homologs and the cytochrome P450 gene family (Figure 9 A–C). Disease resistance gene homologs, most of which have nucleotide binding site-leucine rich repeat (NBS-LRR) domains, express at different levels and tissue specificities, and function in diverse biological processes in Arabidopsis [75]. P450s also express in many tissues in a tissue specific manner and are involved in diverse metabolic processes [76], [77]. The cytochrome P450 family also shows enrichment for DNA based transposed duplications in rice. Thus, these two gene families may have achieved functional and expression diversity through some combination of transposition activity and retention of distantly transposed duplicates. Interestingly, these two families are also enriched with proximal duplications, again often associated with greater expression divergence than WGD despite generally similar coding sequence divergence.

WGD duplicates are enriched in other gene families, such as the cytoplasmic ribosomal protein gene family, and C2H2 zinc finger proteins (Figure 9 D–F). In Arabidopsis, a large number of ribosomal genes are co-regulated [78]. C2H2 zinc finger proteins have been shown to be involved in some basic biological processes such as transcriptional regulation, RNA metabolism and chromatin-remodeling [79]. Furthermore, C2H2 zinc finger proteins are enriched with retained WGD duplicates in both Arabidopsis and rice. Our analyses suggest that gene family members may have common non-random patterns of origin, that recur independently in different evolutionary lineages (such as monocots, and dicots, studied here), and that such patterns may result from specific biological functions and evolutionary needs.

## Discussion

In two species that sample a wide range of tissues and physiological conditions in major angiosperm lineages diverged by about 140–170 million years [80] and affected by at least 5 different genome duplication events, we have compared expression divergence between positional orthologs and between genes duplicated by several additional mechanisms. Both neo-functionalization and genetic redundancy can result in retention of duplicate genes. WGD duplicates generally are more frequently associated with genetic redundancy than genes resulting from other duplication modes, partly due to dosage amplification. Tandem duplications also contribute to genetic redundancy, while other duplication modes are more frequently associated with evolutionary novelty. Potentially transposon mediated gene duplications tend to reduce gene expression levels. Expression divergence between duplicates is discernibly related to duplication modes, WGD events, Ka, Ks, and possibly the DNA methylation status of their promoter regions. However, the contribution of each factor is heterogeneous among duplication modes, and new factors as well as combinatorial effects of different factors are worth further investigation. Gene loss may retard inter-species expression divergence, as singletons are generally more conserved in gene expression than duplicates. Members of different gene families have non-random patterns of origin, and such patterns may be similar between Arabidopsis and rice.

The use of large volumes of data and inclusion of as many genes as possible may help to mitigate factors specific to particular developmental states, noise associated with microarray data, and bias reflecting features specific to particular gene families. For example, we have found that the correlations between expression divergence and Ks are not consistent within gene duplication modes (Figure 5 and 8). For WGD duplicates, significant correlations only exist in those generated by recent WGD events - if only relatively ‘young’ WGD duplicates are studied, the correlations may be overestimated. Moreover, such correlations are not uniformly distributed among Ks levels - at low Ks levels (<1), all duplication modes may show correlations.

We find evidence for duplicate gene retention by both neo-functionalization and genetic redundancy, seemingly at opposite ends of the spectrum of possible fates of duplicated gene pairs. Genetic redundancy has clear biological significance, i.e. provision of buffering capacity [10], [11] and/or dosage balance [34], [46], [47], [48], and seems most closely related to WGD or tandem duplicates. The origins of genetic novelty, of clear biological significance in occupation of new niches or adaptation to new environments, may lie more with the greater expression divergence and more independent evolution of distantly transposed and dispersed duplications. Proximal duplication is more balanced in its contributions to genetic novelty and redundancy than other gene duplication modes.

Detailed delineation of gene duplication modes reveals some new trends. Prior studies classified genes into as few as two types (anchors generated by polyploidy, and non-anchors generated by single gene duplication [58]), or as many as three types (segmental, tandem and dispersed: [38]). In this study, we have attempted to distinguish DNA/RNA based transposed from dispersed duplication, and proximal from tandem duplication. DNA based transposed duplications tend to evolve faster in expression while having smaller Ks than dispersed duplicates. Tandem duplicates diverge slower in gene expression than proximal duplicates. Proximal duplicates tend to diverge faster in expression than WGD duplicates, though concerted evolution [20] may homogenize their coding sequences.

### The factors that affect expression divergence are complex

Our analyses suggest that it may be inappropriate to make generalizations about levels and patterns of expression divergence across gene duplication modes. Ks, putatively a proxy for age, seems to be related to expression divergence only within a subset of duplication modes and largely only among younger duplicates. Ka, putatively a proxy for functional change, also shows statistically significant and heterogeneous relationships to expression divergence. The level of these correlations is very low, even in recent WGD duplicates.

Although expression divergence between duplicates is often significantly correlated with coding sequence divergence, it is well known that gene expression is also regulated by other genomic regions such as promoters, 5′UTRs, and 3′UTRs. The correlations between expression divergence and nucleotide substitution rates (μ) of different genomic regions for pairs of duplicates are summarized in Table S3. WGD duplicates show significant correlations between expression divergence and nucleotide substitution rates in all three regions. These correlations become marginal and often non-significant among tandem duplicates. Expression divergence of proximal duplicates is more closely associated with divergence in promoters, 5′UTRs and 3′UTRs than coding sequences. Expression divergence of DNA based transposed duplicates seem to be most related to Ka and μ of 3′UTRs. Expression divergence of dispersed duplicates is very slightly correlated with Ka but not with other substitution rates. Retrotransposed duplication is least related to any type of sequence divergence, consistent with its general separation of a gene from its native regulatory elements.

In partial summary, expression divergence between duplicate genes may be affected by different and multiple genetic factors depending on the causal duplication mechanism. For pairs of orthologs between Arabidopsis and rice, expression divergence seems only correlated with Ka (Table 5 and Table S3). Single gene duplications including translocated and tandem/proximal duplications have been suggested to be much more prone to promoter disruption than WGD [58]. We examined this hypothesis using >45% sequence identity as criterion for determining duplicated (non-disrupted) promoter regions, finding proximal duplicates to have higher proportions of duplicated promoter regions than WGD duplicates (Table 9). This finding seems to contradict the greater expression divergence of proximal duplicates than WGD duplicates. Thus, we note that each of the investigated genetic/epi-genetic factors may only explain a small portion of the variation of expression divergence between duplicate genes, and perhaps only for certain duplication modes. New factors that may affect expression divergence and how different factors work together are worth investigation.

**Table 9:** Proportion of copied promoter regions among duplicates.

Species	WGD	Tandem duplication	Proximal duplication	DNA based transposed duplication	Retrotransposed duplication	Dispersed duplication
Arabidopsis	0.899	0.923	0.927	0.885	0.865	0.871
Rice	0.382	0.431	0.407	0.344	0.327	0.330

### Possible non-random associations between duplication mode and population size

WGD is often associated with speciation in plants [81], [82]. If ancestral polyploidy was attendant with speciation, new species would have likely initially faced very small *N_e_* (i.e. effective population size), weak selection, high drift and high mutational load. This could put a premium on buffering, but allow little chance for beneficial mutations. On the other hand, small-scale duplications may have been only infrequently associated with speciation, if at all. Thus they might be more likely to arise in established populations with larger *N_e_* and more efficient selection, all putting a greater premium on evolutionary novelty to attain fixation. A hypothesis worthy of further investigation is that non-random associations between duplication mode and population size have shaped which specific genes and functional variations are retained.

## Methods

### Genome annotation

Genome annotations were obtained from TAIR (http://www.arabidopsis.org) for Arabidopsis, and from the Rice Genome Annotation Project data (http://rice.plantbiology.msu.edu) for rice. Gene structures were retrieved using ENSEMBL Biomart (http://plants.ensembl.org/biomart/martview).

### Gene expression data

To reliably assess the expression divergence between duplicates or between orthologs, we used as many publicly available microarray datasets as possible, all of which were obtained from NCBI's GEO (http://www.ncbi.nlm.nih.gov/geo/). At the time of retrieval, 6,009 samples existed for the Affymetrix Arabidopsis ATH1 Genome Array (GEO platform GPL198), of which 800 were not available and a total of 5,209 CEL files were downloaded. 550 CEL files for the Affymetrix GeneChip Rice Genome Array (GEO platform GPL2020) were downloaded, of which 13 were removed due to incorrect array types. For both Arabidopsis and rice raw expression data, RMA normalization was performed using the RMAExpress software (http://rmaexpress.bmbolstad.com) across the entire dataset. Outliers were detected using the arrayQualityMetrics [83] Bioconductor package, which implements three different statistical tests to identify outliers. A total of 443 and 29 samples were detected as outliers and removed in Arabidopsis and rice respectively. Thus, 4,566 and 508 samples remained for Arabidopsis and rice, respectively. The annotation files (Release 30) of these two arrays were downloaded from the Affymetrix website (http://www.affymetrix.com), containing 22,810 Arabidopsis genes and 27,910 rice genes. For a gene, there may be multiple probe sets or multiple types of probe sets available on the array. However, a general rule for selection of a probe set that best represents the gene's expression profile has not been resolved yet [84], [85]. In this study, inclusion or exclusion of “sub-optimal” probe sets with suffix “_s_at” or “_x_at” that are suspected of potential cross-hybridization (may be not sub-optimal in practice according to ref. [84], [85]) had only trivial effects. Thus, to survey as many genes as possible, all types of probe sets were considered, and for a gene with multiple probe sets, we used the first probe set according to alphabetic sorting to represent its expression profile.

### Analysis of expression data

Similarity between the expression profiles of two duplicate genes within species was initially measured by either Pearson's (denoted by PCC or *r*) or Spearman's correlation coefficient. Note that all replicate chips were retained and correlations were computed across all individual chips. These two measures generated highly consistent results, and thus we only showed the statistics measured by Pearson's correlation coefficient. The expression divergence between two duplicate genes or orthologs was measured by 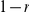 [61], [62].

Orthologous gene pairs compared between Arabidopsis and rice were restricted to 2,012 pairs of orthologs located at corresponding loci in paired syntenic blocks between Arabidopsis and rice as identified by MCScan [53], and having expression profiles on the arrays. To assess the expression conservation (EC) for a pair of Arabidopsis-rice orthologs, we adopted a conceptual framework of comparing co-expression patterns across species [69] implemented in several other studies similar to ours [86], [87], [88], [89], [90]. In this study, the framework can be described as:

1. The expression matrices, **A** and **B**, in Arabidopsis and rice respectively, are restricted to genes for which orthology relationships have been identified and ordered accordingly (i.e., equivalent rows of the two matrices correspond to the expression profiles of a pair of orthologs):
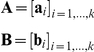
where 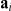 and 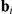 are the vectors of expression profiles for any pair *i* of orthologs for Arabidopsis and rice, respectively, and *k* is the number of orthologous gene pairs.
2. **A** and **B** are then converted into two pair-wise correlation matrices, 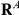 and 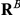, by computing the PCCs between the expression profile of each gene and that of any other gene in each species separately:
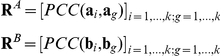

3. The expression conservation for an orthologous gene pair *i* is computed as:
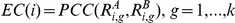
Its corresponding expression divergence is 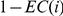.

### Identification of different modes of gene duplications

The populations of potential gene duplications in Arabidopsis or rice were identified using BLASTP. Only the top five non-self protein matches that met a threshold of 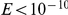 were considered. Genes without BLASTP hits that met a threshold of 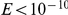 were deemed singletons. Pairs of WGD duplicates were downloaded from the PGDD database [51], [53]. Pairs of α, β, γ duplicates in Arabidopsis and pairs of ρ, σ duplicates in rice were obtained from published lists [49], [54]. Single gene duplications were derived by excluding pairs of WGD duplicates from the population of gene duplications. Tandem duplications were defined as being adjacent to each other on the same chromosome. Proximal duplications were defined as non-tandem genes within 20 annotated genes of each other on the same chromosome [38].

The remaining single gene duplications (after deducting tandem and proximal duplications) were searched for distant single gene-transposed duplications. To accomplish this aim, genes at ancestral chromosomal positions need to be discerned by aligning syntenic blocks within and between species [53], [55]. Angiosperm syntenic blocks were downloaded from the Plant Genome Duplication Database (PGDD), available at http://chibba.agtec.uga.edu/duplication. At the time of retrieval, PGDD provided syntenic blocks within and between 10 species including *Arabidopsis thaliana, Carica papaya, Prunus persica, Populus trichocarpa, Medicago truncatula, Glycine max, Vitis vinifera, Brachypodium distachyon, Oryza sativa, Sorghum bicolor, Zea mays* [51], [53]. An Arabidopsis or rice gene locus was regarded as ancestral if the resident gene along with any of its homologous genes (paralogs/orthologs) occur at corresponding loci within any pair of syntenic blocks in PGDD. Using this criterion, the population of Arabidopsis/rice genes was divided into two subsets: genes at ancestral loci and genes that were transposed. For a pair of distantly transposed duplicate genes, we required that one copy was at its ancestral locus and the other was at a non-ancestral locus, named the parental copy and transposed copy respectively. If the parental copy has more than two exons and the transposed copy is intronless, we inferred that this pair of duplicate genes occurred by retrotransposition (RNA based transposition). If both copies have a single exon, the pair of duplicates was unclassified. For other cases of a pair of distantly transposed duplicate genes, we inferred that the duplication occurred by DNA based transposition. The remaining single gene duplications in the population, i.e. after deducting WGD, tandem, proximal, DNA based transposed and retrotransposed duplications from the BLASTP output, were classified as dispersed duplications. After pairs of duplicate genes in each duplication mode were identified, we assigned a unique origin to each duplicated gene, according to the following order of priority: WGD>tandem>proximal>retrotransposed>DNA based transposed>dispersed.

### GO/Pfam enrichment analysis

GO/Pfam enrichment analysis was performed using Fisher's exact test. The *P*-value was calculated for the null hypothesis that there is no association between a subset of genes and a particular functional/domain category and was corrected with the total number of terms to account for multiple comparisons.

### Assessing DNA sequence divergence

Coding sequence divergence between a pair of genes was denoted by either non-synonymous (Ka) or synonymous (Ks) substitution rates. Protein sequences were aligned using Clustalw [91] with default parameters. The protein alignment was then converted to DNA alignment using the “Bio::Align::Utilities” module of the BioPerl package (http://www.bioperl.org/). Ka and Ks were estimated by Nei-Gojobori statistics [92], available through the “Bio::Align::DNAStatistics” module of the BioPerl package. Note that the “Bio::Align::DNAStatistics” module may generate invalid Ka or Ks for some duplicate gene pairs due to mis-alignments, which were ruled out from related analysis. All levels of valid Ka or Ks values were considered in related statistical analyses. Because distributions of Ka or Ks were centered at low levels (∼1.0), in related figures, to improve their clarity, we only displayed Ka or Ks values between 0 and 2.0.

The promoter region of a gene was restricted to a maximum of 1,000 bp upstream of the transcription start site (TSS) or less if the nearest adjacent upstream gene is closer than 1,000 bp. For a pair of genes, the divergence of promoter sequences was indicated by their Jukes-Cantor nucleotide substitution rate (μ) [93], which is available through the “Bio::Align::DNAStatistics” module of the BioPerl package. The divergence in 5′UTR and 3′UTR is also measured by nucleotide substitution rates (μ). Note that the “Bio::Align::DNAStatistics” module may not output μ if the distance between two input nucleotide sequences is too near or too far. Duplicate gene pairs lacking estimation of μ in the promoter region, 5′UTR or 3′UTR were removed from related analysis.

### DNA methylation data and its analysis

Arabidopsis and rice genome-wide DNA methylation data were obtained from GEO (accession number: GSE21152) [73]. We chose this study, which provided DNA methylation for both Arabidopsis and rice, because the systematic errors between species should be smaller than in data from separate studies. A gene methylated in the promoter region is defined by the presence of two or more adjacent methylated probes within the promoter DNA sequence [59], [72].

### Gene families

Lists of published gene families were obtained from TAIR (http://www.arabidopsis.org/browse/genefamily/index.jsp) for Arabidopsis, and from the Rice Genome Annotation Project data (http://rice.plantbiology.msu.edu/annotation_community_families.shtml) for rice. Only families with more than nine genes were considered. Arabidopsis disease resistance gene homologs were downloaded from the NIBLRRS Project website (http://niblrrs.ucdavis.edu/). The Rice Cytochrome P450 gene family was downloaded from the Cytochrome P450 homepage [94] .

## Supporting Information

Table S1 **Enriched GO terms and Pfam domains associated with the duplicates of conserved or divergent expression at each WGD event.**
(DOCX)

Table S2 **List of investigated gene families and their enrichments with modes of gene duplication.**
(DOCX)

Table S3 **Correlations between expression divergence and different types of sequence divergence.**
(DOCX)
