# Supplementary material for: Modes of Gene Duplication Contribute Differently to Genetic Novelty and Redundancy, but Show Parallels across Divergent Angiosperms
Source: PLoS One. 2011 Dec 2;6(12):e28150. doi: 10.1371/journal.pone.0028150 (PMC3229532; doi:10.1371/journal.pone.0028150)
Supplement: Table S1 — Enriched GO terms and Pfam domains associated with the duplicates of conserved or divergent expression at each WGD event. (DOCX) [file pone.0028150.s001.docx]

**Table S1. Enriched GO terms and Pfam domains associated with the duplicates of conserved or divergent expression at each WGD event**

| WGD event | GO terms | | Pfam domains | |
| --- | --- | --- | --- | --- |
|  | Conserved expression | Divergent expression | Conserved expression | Divergent expression |
| Arabidopsis |  |  |  |  |
| α | GO:0003735, structural constituent of ribosome (F);  GO:0006412, protein biosynthesis (P);  GO:0005840, ribosome (C);  GO:0005730, nucleolus (C);  GO:0042254, ribosome biogenesis and assembly (P);  GO:0009507, chloroplast (C);  GO:0009570, chloroplast stroma (C);  GO:0009579, thylakoid (C);  GO:0005634, nucleus(C);  GO:0003700, transcription factor activity (F);  GO:0009535, thylakoid membrane (sensu Viridiplantae) (C);  GO:0003677, DNA binding (F);  GO:0015934, large ribosomal subunit (C);  GO:0005839 ,proteasome core complex (sensu Eukaryota) (C);  GO:0010200, response to chitin (P);  GO:0015979, photosynthesis (P);  GO:0005618, cell wall (C); GO:0006334, nucleosome assembly (P);  GO:0045449, regulation of transcription (P) | GO:0003824, catalytic activity (F);  GO:0016491, oxidoreductase activity (F);  GO:0012505, endomembrane system (C);  GO:0006952, defense response (P);  GO:0008152, metabolism(P);  GO:0016787, hydrolase activity (F);  GO:0004126, cytidine deaminase activity (F);  GO:0005351, sugar porter activity (F);  GO:0005576, extracellular region (C);  GO:0016788, hydrolase activity, acting on ester bonds (F) | PF00227, proteasome;  PF01423, Sm | PF00450, serine_carbpept;  PF01657,DUF26 |
| β | GO:0006412, protein biosynthesis (P);  GO:0003735, structural constituent of ribosome (F);  GO:0005840 , ribosome(C);  GO:0042254 , ribosome biogenesis and assembly (P); GO:0005730, nucleolus(C) | - | PF02309, AUX_IAA;  PF02365, NAM;  PF00394, Cu-oxidase | PF00702, Hydrolase |
| γ | GO:0003677, DNA binding(F) ;  GO:0003700, transcription factor activity (F) | GO:0015144, carbohydrate transporter activity(F) | - | - |
| Rice |  |  |  |  |
| ρ | GO:0005840, ribosome (C);  GO:0003700, transcription factor activity (F);  GO:0006350, transcription (P);  GO:0005198, structural molecule activity (F);  GO:0006412 , protein biosynthesis (P);  GO:0007275, development (P);  GO:0005634, nucleus (C);  GO:0003677 , DNA binding (F);  GO:0016043, cell organization and biogenesis (P);  GO:0005829, cytosol (C) | GO:0005215, transporter activity (F);  GO:0005783, endoplasmic reticulum (C);  GO:0005773, vacuole(C);  GO:0005739, mitochondrion (C) ;  GO:0006810, transport (P); GO:0006519, amino acid and derivative metabolism (P);  GO:0016020 , membrane (C);  GO:0009058 , biosynthesis (P) | PF02365, NAM;  PF00249, Myb_DNA-binding;  PF01095, Pectinesterase  PF00319 SRF-TF | PF00450, Peptidase_S10;  PF00635, Motile_Sperm;  PF00005, ABC_tran |
| σ | GO:0009653, morphogenesis (P);  GO:0030528 , transcription regulator activity (F) | - | PF04690, YABBY;  PF00010, HLH | - |
