# Supplementary material for: Modes of Gene Duplication Contribute Differently to Genetic Novelty and Redundancy, but Show Parallels across Divergent Angiosperms
Source: PLoS One. 2011 Dec 2;6(12):e28150. doi: 10.1371/journal.pone.0028150 (PMC3229532; doi:10.1371/journal.pone.0028150)
Supplement: Table S2 — List of investigated gene families and their enrichments with modes of gene duplication. (DOCX) [file pone.0028150.s002.docx]

**Table S2. List of investigated gene families and their enrichments with modes of gene duplication**

| Species | Gene family | Size | Enrichment | | | | | |
| --- | --- | --- | --- | --- | --- | --- | --- | --- |
|  |  |  | WGD | Tandem | Proximal | DNA based transposed | Retron-transposed | Dispersed |
| Arabidopsis | 14-3-3 proteins | 13 | 0.605 | 1 | 1 | 1 | 1 | 0.258 |
| Arabidopsis | AAAP family | 42 | 1 | 1 | 1 | 1 | 1 | 6.21E-05 |
| Arabidopsis | ABC Superfamily | 123 | 1 | 0.827 | 0.132 | 0.022 | 1 | 1 |
| Arabidopsis | ABC transporters | 93 | 1 | 0.442 | 0.055 | 0.517 | 1 | 1 |
| Arabidopsis | ABC transporters (Smart Lab) | 12 | 1 | 1 | 1 | 0.872 | 1 | 1 |
| Arabidopsis | ABI3VP1 Transcription Factor Family | 11 | 1 | 1 | 1 | 0.078 | 1 | 1 |
| Arabidopsis | ACA | 13 | 1 | 1 | 1 | 1 | 1 | 0.132 |
| Arabidopsis | Acyl Lipid Metabolism Family | 602 | 0.093 | 0.046 | 1 | 1 | 1 | 1 |
| Arabidopsis | AGC Family | 39 | 1 | 1 | 1 | 1 | 1 | 5.82E-05 |
| Arabidopsis | Aldehyde Dehydrogenase Superfamily | 14 | 1 | 1 | 1 | 1 | 1 | 4.00E-04 |
| Arabidopsis | Antiporter Superfamily | 28 | 1 | 0.187 | 1 | 1 | 1 | 1 |
| Arabidopsis | Antiporters | 68 | 1 | 0.387 | 1 | 1 | 1 | 1 |
| Arabidopsis | AP2-EREBP Transcription Factor Family | 135 | 0.266 | 1 | 1 | 1 | 1 | 4.75E-12 |
| Arabidopsis | Aquaporin Families | 34 | 0.286 | 1 | 1 | 0.154 | 1 | 1 |
| Arabidopsis | ARF Transcription Factor Family | 21 | 1 | 1 | 1 | 0.127 | 1 | 1 |
| Arabidopsis | ARIADNE gene family | 15 | 1 | 0.274 | 1 | 1.70E-03 | 1 | 1 |
| Arabidopsis | ARR-B Transcription Factor Family | 15 | 1 | 1 | 1 | 1 | 1 | 1.52E-05 |
| Arabidopsis | AS2 family | 42 | 1 | 1 | 1 | 1 | 1 | 0.541 |
| Arabidopsis | basic Helix-Loop-Helix (bHLH) Transcription Factor | 160 | 0.479 | 1 | 1 | 1 | 1 | 8.66E-03 |
| Arabidopsis | basic region leucine zipper (bZIP) Transcription Factor | 72 | 0.121 | 1 | 1 | 1 | 1 | 1 |
| Arabidopsis | bHLH Transcription Factor Family | 158 | 0.479 | 1 | 1 | 1 | 1 | 8.66E-03 |
| Arabidopsis | bZIP Transcription Factor Family | 70 | 0.102 | 1 | 1 | 1 | 1 | 1 |
| Arabidopsis | C2C2-CO-like Transcription Factor Family | 29 | 0.069 | 1 | 1 | 1 | 1 | 1 |
| Arabidopsis | C2C2-Dof Transcription Factor Family | 36 | 0.028 | 1 | 1 | 1 | 1 | 0.208 |
| Arabidopsis | C2C2-Gata Transcription Factor Family | 30 | 2.97E-03 | 1 | 1 | 1 | 1 | 1 |
| Arabidopsis | C2H2 Transcription Factor Family | 207 | 1.41E-04 | 1 | 1 | 1 | 1 | 1 |
| Arabidopsis | C3H Transcription Factor Family | 162 | 0.099 | 1 | 1 | 1 | 1 | 0.023 |
| Arabidopsis | Calcium Dependent Protein Kinase | 34 | 0.091 | 1 | 1 | 1 | 1 | 0.805 |
| Arabidopsis | Carbohydrate Esterase Gene Families | 85 | 1 | 1 | 1 | 1 | 1 | 0.454 |
| Arabidopsis | CBL-interacting serione-threonine Protein Kinases (AtCIPKs) | 25 | 0.431 | 1 | 1 | 1 | 1 | 0.35 |
| Arabidopsis | CCAAT-HAP2 Transcription Factor Family | 10 | 1 | 1 | 1 | 1 | 1 | 0.154 |
| Arabidopsis | CCAAT-HAP3 Transcription Factor Family | 10 | 1 | 1 | 1 | 1 | 1 | 1 |
| Arabidopsis | CCAAT-HAP5 Transcription Factor Family | 13 | 1 | 1 | 1 | 1 | 1 | 1 |
| Arabidopsis | CDPKs | 33 | 0.102 | 1 | 1 | 1 | 1 | 0.285 |
| Arabidopsis | Chloroplast and Mitochondria gene families | 49 | 1 | 1 | 1 | 1 | 1 | 1 |
| Arabidopsis | Class III peroxidase | 73 | 1 | 0.796 | 1 | 1 | 1 | 1 |
| Arabidopsis | CNGCs | 20 | 1 | 1 | 1 | 1 | 1 | 1 |
| Arabidopsis | COBRA Gene Family | 11 | 1 | 1 | 1 | 1 | 1 | 0.073 |
| Arabidopsis | Core Cell Cycle Genes | 59 | 0.147 | 1 | 1 | 1 | 0.15 | 0.621 |
| Arabidopsis | Core DNA replication machinery | 61 | 1 | 1 | 1 | 2.96E-05 | 7.39E-03 | 1 |
| Arabidopsis | Cytochrome P450 | 219 | 1 | 2.55E-09 | 2.39E-18 | 6.84E-11 | 1 | 1 |
| Arabidopsis | Cytoplasmic ribosomal protein gene family | 233 | 7.42E-30 | 1 | 1 | 0.036 | 0.975 | 1 |
| Arabidopsis | Cytoskeleton | 12 | 0.337 | 1 | 1 | 1 | 1 | 1 |
| Arabidopsis | Disease Resistance Gene Homologs | 188 | 1 | 0.19 | 4.94E-18 | 2.71E-34 | 1 | 1 |
| Arabidopsis | Dof family | 36 | 0.028 | 1 | 1 | 1 | 1 | 0.208 |
| Arabidopsis | EF-hand containing proteins | 188 | 0.175 | 1 | 1 | 1 | 1 | 0.26 |
| Arabidopsis | Eukaryotic Initiation Factor Gene Family | 93 | 0.028 | 1 | 1 | 0.531 | 0.017 | 1 |
| Arabidopsis | EXO70 exocyst subunit family | 23 | 1 | 1 | 1 | 1 | 1 | 1 |
| Arabidopsis | Expansins | 34 | 0.458 | 1 | 0.285 | 1 | 1 | 1 |
| Arabidopsis | Family of Arabidopsis genes related to Xyloglucan Fucosyltransferase1 | 12 | 1 | 0.122 | 1 | 1 | 1 | 1 |
| Arabidopsis | FH2 proteins | 20 | 1 | 1 | 1 | 0.184 | 1 | 1 |
| Arabidopsis | FtsH: AAA ATP-dependent zinc metallopeptidase | 12 | 1 | 1 | 1 | 0.026 | 1 | 1 |
| Arabidopsis | G2-like Transcription Factor Family | 40 | 1 | 1 | 1 | 0.149 | 1 | 1 |
| Arabidopsis | GeBP Transcription Factor Family | 16 | 1 | 1 | 1 | 1 | 1 | 1 |
| Arabidopsis | Glutathione S-transferase Family | 53 | 1.71E-03 | 2.83E-05 | 2.79E-07 | 0.38 | 1 | 1 |
| Arabidopsis | Glycoside Hydrolase Gene Families | 364 | 1 | 0.019 | 0.418 | 7.18E-10 | 1 | 1 |
| Arabidopsis | Glycosyltransferase Gene Families | 317 | 1 | 5.97E-03 | 2.53E-03 | 1 | 1 | 1 |
| Arabidopsis | GRAS Gene Family | 32 | 0.723 | 1 | 1 | 1 | 1 | 3.66E-04 |
| Arabidopsis | GRAS Transcription Factor Family | 31 | 0.509 | 1 | 1 | 1 | 1 | 1.23E-03 |
| Arabidopsis | Heat Shock Transcription Factor Family | 21 | 1 | 1 | 1 | 1 | 1 | 2.39E-08 |
| Arabidopsis | Histidine Kinase | 16 | 1 | 1 | 1 | 1 | 1 | 0.023 |
| Arabidopsis | Homeobox Transcription Factor Family | 91 | 0.34 | 1 | 1 | 1 | 1 | 1.08E-09 |
| Arabidopsis | HSF Transcription Factor Family | 21 | 1 | 1 | 1 | 1 | 1 | 2.39E-08 |
| Arabidopsis | IDZ Gene Family | 11 | 1 | 1 | 1 | 1 | 1 | 0.073 |
| Arabidopsis | Inorganic Solute Cotransporters | 83 | 1 | 1 | 1 | 1 | 1 | 1 |
| Arabidopsis | Ion Channel Families | 61 | 1 | 1 | 1 | 0.018 | 1 | 1 |
| Arabidopsis | IQD Protein Family | 33 | 1 | 1 | 1 | 1 | 1 | 8.35E-03 |
| Arabidopsis | Kinesins | 61 | 1 | 1 | 1 | 1 | 1 | 3.70E-04 |
| Arabidopsis | Lateral Organ Boundaries Gene Family | 42 | 1 | 1 | 1 | 1 | 1 | 0.541 |
| Arabidopsis | Leucine-rich repeat extensin | 11 | 1 | 1 | 1 | 1 | 1 | 0.281 |
| Arabidopsis | Lipid Metabolism Gene Families | 96 | 1 | 1 | 1 | 1 | 1 | 1 |
| Arabidopsis | MADS like gene family | 16 | 1 | 1 | 0.032 | 1 | 1 | 0.141 |
| Arabidopsis | MADS Transcription Factor Family | 105 | 1 | 1 | 1 | 1 | 1 | 0.07 |
| Arabidopsis | MADS-box Transcription Factor Family | 106 | 1 | 1 | 0.822 | 1 | 1 | 0.031 |
| Arabidopsis | Magnesium Transporter Gene Family | 11 | 1 | 1 | 1 | 1 | 1 | 1 |
| Arabidopsis | MAP Kinase (MAPK) Family | 20 | 1 | 1 | 1 | 1 | 1 | 6.95E-03 |
| Arabidopsis | MAP Kinase Kinase (MAPKK) Family | 10 | 1 | 1 | 1 | 1 | 1 | 0.038 |
| Arabidopsis | MAP Kinase Kinase Kinase (MAPKKK) Family | 80 | 1 | 1 | 1 | 1 | 1 | 4.40E-08 |
| Arabidopsis | MAP Kinase Kinase Kinase Kinase (MAPKKKK) Family | 10 | 1 | 1 | 1 | 1 | 1 | 0.198 |
| Arabidopsis | Mechanosensitive Ion Channel Family | 10 | 1 | 1 | 1 | 1 | 1 | 1 |
| Arabidopsis | MIP family | 37 | 0.209 | 1 | 1 | 0.205 | 1 | 1 |
| Arabidopsis | Miscellaneous Membrane Protein Families | 438 | 1 | 1 | 1 | 0.018 | 1 | 1 |
| Arabidopsis | MLO proteins | 14 | 1 | 1 | 1 | 0.493 | 1 | 1 |
| Arabidopsis | Monolignol Biosynthesis | 61 | 1 | 0.017 | 1 | 1.84E-04 | 0.058 | 1 |
| Arabidopsis | Monosaccharide transporter-like gene family | 53 | 0.82 | 1 | 1 | 1 | 1 | 0.532 |
| Arabidopsis | MYB | 131 | 0.015 | 1 | 1 | 1 | 1 | 7.08E-06 |
| Arabidopsis | MYB Transcription Factor Family | 130 | 0.011 | 1 | 1 | 1 | 1 | 1.40E-05 |
| Arabidopsis | Myosin | 17 | 0.937 | 1 | 1 | 1 | 1 | 0.101 |
| Arabidopsis | NAC Transcription Factor Family | 93 | 1 | 1 | 1 | 1 | 0.144 | 5.73E-03 |
| Arabidopsis | Nodulin-like protein family | 63 | 1 | 1 | 1 | 1 | 1 | 0.029 |
| Arabidopsis | Nucleobase ascorbate transporters, NAT family | 12 | 1 | 1 | 1 | 1 | 1 | 9.06E-03 |
| Arabidopsis | Organic Solute Cotransporters | 273 | 1 | 1 | 1 | 1 | 1 | 3.59E-03 |
| Arabidopsis | PHD Transcription Factor Family | 11 | 1 | 1 | 1 | 0.112 | 1 | 1 |
| Arabidopsis | Phospholipase D | 12 | 1 | 1 | 1 | 0.245 | 1 | 1 |
| Arabidopsis | Phospholipase D (Zarsky group) | 11 | 1 | 1 | 1 | 0.121 | 1 | 1 |
| Arabidopsis | Phosphoribosyltransferases (PRT) | 15 | 0.25 | 1 | 1 | 1 | 1.89E-04 | 1 |
| Arabidopsis | Plant Cell Wall Biosynthesis Families | 30 | 1 | 1 | 1 | 1 | 1 | 1 |
| Arabidopsis | Plant defensins superfamily | 15 | 1 | 0.042 | 1 | 0.338 | 1 | 1 |
| Arabidopsis | Plant U-box protein (PUB) | 61 | 1 | 1 | 1 | 1 | 1 | 0.03 |
| Arabidopsis | Polysaccharide Lyase Gene Families | 30 | 1 | 1 | 1 | 1 | 1 | 1 |
| Arabidopsis | PP2C-type phosphatases | 76 | 1 | 1 | 1 | 1 | 1 | 6.93E-06 |
| Arabidopsis | Primary Pumps (ATPases) Gene Families | 79 | 3.51E-04 | 1 | 1 | 0.683 | 1 | 1 |
| Arabidopsis | Primary Pumps (ATPases) Gene Family (2) | 32 | 1.40E-05 | 1 | 1 | 1 | 1 | 1 |
| Arabidopsis | Protein tyrosine phosphatase (PTP) family | 28 | 1 | 1 | 1 | 1 | 0.113 | 1 |
| Arabidopsis | Rad5/16-like gene family | 10 | 1 | 1 | 1 | 1 | 1 | 8.60E-04 |
| Arabidopsis | RAV Transcription Factor Family | 11 | 0.2 | 1 | 1 | 1 | 1 | 1 |
| Arabidopsis | Receptor kinase-like protein family | 302 | 0.786 | 0.049 | 4.90E-17 | 1 | 1 | 1 |
| Arabidopsis | REM Transcription Factor Family | 20 | 1 | 0.412 | 2.98E-13 | 1 | 1 | 1 |
| Arabidopsis | Response Regulator | 32 | 1 | 1 | 1 | 1 | 1 | 2.60E-08 |
| Arabidopsis | SBP Transcription Factor Family | 16 | 1 | 1 | 1 | 1 | 1 | 5.98E-03 |
| Arabidopsis | Single gene-encoded CBPs | 11 | 1 | 1 | 1 | 1 | 1 | 1 |
| Arabidopsis | SNARE Interacting Proteins | 13 | 0.963 | 1 | 1 | 1 | 1 | 1 |
| Arabidopsis | SNAREs | 54 | 0.018 | 1 | 1 | 1 | 1 | 1 |
| Arabidopsis | Subtilisin-like Serine Proteases | 54 | 1 | 0.021 | 0.248 | 1 | 1 | 1 |
| Arabidopsis | Sulfurtransferasese / Rhodanese Family | 18 | 1 | 1 | 1 | 1 | 1 | 1 |
| Arabidopsis | Superfamily of zinc-coordinating DNA-binding proteins | 17 | 1 | 1 | 1 | 1 | 1 | 0.085 |
| Arabidopsis | tify family | 18 | 0.089 | 1 | 1 | 1 | 1 | 1 |
| Arabidopsis | Trehalose Biosynthesis Gene Families | 12 | 1 | 1 | 1 | 1 | 1 | 0.799 |
| Arabidopsis | Trihelix Transcription Factor Family | 28 | 0.23 | 1 | 1 | 1 | 1 | 0.64 |
| Arabidopsis | TUB Transcription Factor Family | 10 | 1 | 1 | 1 | 1 | 1 | 0.025 |
| Arabidopsis | Type I MADS box | 42 | 1 | 1 | 1 | 0.156 | 1 | 1 |
| Arabidopsis | WOX gene family | 15 | 1 | 1 | 1 | 1 | 1 | 7.04E-03 |
| Arabidopsis | WRKY Transcription Factor Family | 71 | 1 | 1 | 1 | 1 | 1 | 1.17E-07 |
| Arabidopsis | WRKY Transcription Factor Superfamily | 69 | 1 | 1 | 1 | 1 | 1 | 1.67E-06 |
| Arabidopsis | ZF-HD Transcription Factor Family | 15 | 0.698 | 1 | 1 | 1 | 1 | 0.052 |
| Arabidopsis | zinc finger-homeobox gene family | 17 | 0.867 | 1 | 1 | 1 | 1 | 0.014 |
| rice | BTB | 105 | 1 | 1 | 4.54E-05 | 1 | 0.366 | 1 |
| rice | C2H2 | 141 | 0.042 | 1 | 1 | 1 | 1 | 1.93E-06 |
| rice | Calmodulin | 33 | 8.36E-05 | 1 | 1 | 1 | 1 | 0.012 |
| rice | Cellulose Synthase | 42 | 1 | 1 | 1 | 1 | 1 | 0.624 |
| rice | Conserved peptide uORF-containing transcripts | 35 | 0.083 | 1 | 1 | 1 | 1 | 1 |
| rice | Core Replication Machinery Proteins | 57 | 1 | 1 | 1 | 1 | 1 | 1.99E-03 |
| rice | Cysteine Rice Peptides | 329 | 2.24E-03 | 2.04E-19 | 4.89E-26 | 1 | 1 | 1 |
| rice | Early Auxin-responsive AuxIAA | 29 | 0.84 | 1 | 1 | 1 | 1 | 1.15E-07 |
| rice | Early Auxin-responsive GH3 | 13 | 1 | 1 | 1 | 1 | 0.05 | 1 |
| rice | F box | 451 | 1 | 6.59E-07 | 2.52E-22 | 1 | 1 | 1 |
| rice | Glutaredoxin | 23 | 1 | 1 | 1 | 1 | 1 | 0.177 |
| rice | Glycosyl Hydrolase Family 1 Beta-Glucosidases | 35 | 1 | 0.129 | 1 | 4.18E-04 | 0.065 | 1 |
| rice | Indole 3 acetic acid synthetase | 12 | 1 | 1 | 1 | 1 | 0.035 | 1 |
| rice | kinase interactome | 144 | 0.022 | 1 | 1 | 1 | 1 | 1 |
| rice | MADS box | 44 | 0.313 | 1 | 1 | 1 | 1 | 9.25E-05 |
| rice | MAPK | 20 | 0.09 | 1 | 1 | 1 | 0.173 | 1 |
| rice | p450 | 222 | 1 | 0.787 | 7.40E-03 | 8.65E-23 | 1 | 1 |
| rice | PEBP | 12 | 0.219 | 1 | 1 | 0.93 | 1 | 1 |
| rice | Protein Disulfide Isomerase | 19 | 1 | 1 | 1 | 1 | 1 | 5.73E-05 |
| rice | SBP | 18 | 1 | 1 | 1 | 1 | 1 | 2.08E-04 |
| rice | serine proteases | 180 | 1 | 1 | 1 | 1 | 0.795 | 3.22E-03 |
| rice | Small auxin-up RNA | 41 | 0.617 | 0.037 | 1.01E-12 | 1 | 1 | 1 |
| rice | Wall associated kinase | 84 | 1 | 0.124 | 4.04E-06 | 2.69E-04 | 0.37 | 1 |
| rice | WRKY | 59 | 0.192 | 1 | 1 | 1 | 1 | 2.27E-05 |
