# Supplementary material for: Modes of Gene Duplication Contribute Differently to Genetic Novelty and Redundancy, but Show Parallels across Divergent Angiosperms
Source: PLoS One. 2011 Dec 2;6(12):e28150. doi: 10.1371/journal.pone.0028150 (PMC3229532; doi:10.1371/journal.pone.0028150)
Supplement: Table S3 — Correlations between expression divergence and different types of sequence divergence. (DOCX) [file pone.0028150.s003.docx]

**Table S3. Correlations between expression divergence and different types of sequence divergence**

| Species | Type of homologs | Number of valid gene pairs | Pearson correlation^1^ (and P-value) between gene expression divergence and | | | | |
| --- | --- | --- | --- | --- | --- | --- | --- |
|  |  |  | Ka | Ks | µ of promoter region | µ of 3’ UTR | µ of 5’ UTR |
| Arabidopsis | WGD | 2,839 | **0.252** ($<2.2\times10^{-16}$) | **0.209** ($<2.2\times10^{-16}$) | **0.147**  ($3.997\times10^{-15}$) | **0.159** ($<2.2\times10^{-16}$) | **0.124** ($3.681\times10^{-11}$) |
|  | Tandem | 383 | 0.040 (0.434) | 0.187 ($2.289\times10^{-4}$) | 0.263 ($1.717\times10^{-7}$) | 0.260 ($2.451\times10^{-7}$) | 0.256 ($3.992\times10^{-7}$) |
|  | Proximal | 379 | 0.075 (0.144) | 0.129 (0.012) | **0.342** ($8.09\times10^{-12}$) | **0.217** ($2.034\times10^{-5}$) | **0.246 (**$1.196\times10^{-6}$) |
|  | DNA based transposed | 1,483 | **0.059** (0.023) | $4.86\times10^{-4}$ (0.985) | -0.007 (0.795) | **0.057** (0.028) | 0.023 (0.373) |
|  | Retrotransposed | 112 | -0.091 (0.341) | 0.167 (0.079) | 0.020 (0.832) | 0.006 (0.947) | -0.121 (0.204) |
|  | Dispersed | 12,295 | **0.065** ($7.565\times10^{-13}$) | 0.016 (0.073) | 0.022 (0.013) | 0.011 (0.220) | 0.015 (0.092) |
| Rice | WGD | 1,182 | **0.073** (0.012) | **0.119** ($3.86\times10^{-5}$) | **0.159** ($3.859\times10^{-8}$) | **0.099** ($6.712\times10^{-4}$) | **0.073** (0.012) |
|  | Tandem | 203 | 0.011 (0.874) | 0.095 (0.178) | 0.087 (0.219) | -0.011 (0.868) | 0.052 (0.460) |
|  | Proximal | 340 | 0.079 (0.146) | 0.041 (0.445) | **0.017** (0.049) | **0.138** (0.011) | **0.119** (0.029) |
|  | DNA based transposed | 1,720 | **0.059** (0.015) | 0.027 (0.259) | 0.069 (0.004) | **0.108** ($6.089\times10^{-6}$) | 0.096 ($6.702\times10^{-5}$) |
|  | Retrotransposed | 258 | -0.054 (0.386) | 0.018 (0.763) | -0.027 (0.668) | -0.014 (0.821) | -0.010 (0.874) |
|  | Dispersed | 8,549 | **0.032** (0.003) | 0.022 (0.046) | 0.021 (0.054) | 0.025 (0.023) | 0.059 ($4.303\times10^{-8}$) |
| Arabidopsis and rice | Orthologs | 641 | 0.131 ($8.827\times10^{-4}$) | -0.082 (0.037) | 0.046 (0.236) | 0.043 (0.282) | -0.049 (0.214) |

^1^Bold values indicate statistical significance in both Arabidopsis and rice
